# The role of deep learning‐based survival model in improving survival prediction of patients with glioblastoma

**DOI:** 10.1002/cam4.4230

**Published:** 2021-08-28

**Authors:** Hajar Moradmand, Seyed Mahmoud Reza Aghamiri, Reza Ghaderi, Hamid Emami

**Affiliations:** ^1^ Medical Radiation Engineering Shahid Beheshti University Tehran Iran; ^2^ Electrical Engineering Shahid Beheshti University Tehran Iran; ^3^ Department of Radiation Oncology Isfahan University of Medical Sciences Seyed Al‐Shohada Charity Hospital Isfahan Iran

**Keywords:** decision support systems, deep learning, glioblastoma, hyperparameter optimization, survival analysis

## Abstract

This retrospective study has been conducted to validate the performance of deep learning‐based survival models in glioblastoma (GBM) patients alongside the Cox proportional hazards model (CoxPH) and the random survival forest (RSF). Furthermore, the effect of hyperparameters optimization methods on improving the prediction accuracy of deep learning‐based survival models was investigated. Of the 305 cases, 260 GBM patients were included in our analysis based on the following criteria: demographic information (i.e., age, Karnofsky performance score, gender, and race), tumor characteristic (i.e., laterality and location), details of post‐surgical treatment (i.e., time to initiate concurrent chemoradiation therapy, standard treatment, and radiotherapy techniques), and last follow‐up time as well as the molecular markers (i.e., O‐6‐methylguanine methyltransferase and isocitrate dehydrogenase 1 status). Experimental results have demonstrated that age (Elderly > 65: hazard ratio [HR] = 1.63; 95% confidence interval [CI]: 1.213–2.18; *p* value = 0.001) and tumors located at multiple lobes ([HR] = 1.75; 95% [CI]: 1.177–2.61; *p* value = 0.006) were associated with poorer prognosis. In contrast, age (young < 40: [HR] = 0.57; 95% [CI]: 0.343–0.96; *p* value = 0.034) and type of radiotherapy (others include stereotactic and brachytherapy: [HR] = 0.5; 95%[CI]: 0.266–0.95; *p* value = 0.035) were significantly related to better prognosis. Furthermore, the proposed deep learning‐based survival model (concordance index [*c*‐index] = 0.823 configured by Bayesian hyperparameter optimization), outperformed the RSF (*c*‐index = 0.728), and the CoxPH model (*c*‐index = 0.713) in the training dataset. Our results show the ability of deep learning in learning a complex association of risk factors. Moreover, the remarkable performance of the deep‐learning‐based survival model could be promising to support decision‐making systems in personalized medicine for patients with GBM.

## Graphical abstract

This study assesses the performance of deep learning‐based survival models in glioblastoma patients alongside the Cox proportional hazards model and the random survival forests and investigates the effect of hyperparameters optimization methods on improving the prediction accuracy of deep learning‐based survival models. Our results demonstrate the outstanding ability of deep learning in learning a complex association of risk factors.

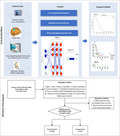

## 1 INTRODUCTION

Glioblastoma (GBM) is the most common fatal malignant brain tumor in adults, with an incidence rate of 3.2 per 100,000 populations.^1^ The current approved treatment of GBM is the maximum safe resection surgery of the tumor with a minimum side effect, followed by the combination of radiotherapy and chemotherapy generally with temozolomide.^2^ The prognosis of GBM patients (median overall survival of 14 months) has remained poor in the past three decades, even with severe multi‐pronged therapies. In some cases (≤10%), a 5‐year survival rate has been reported with the same routine treatment procedure.

Accurate prediction of individual patients’ prognosis is a crucially important task not only for patients and their families but also for physicians to support personalized treatment and to identify who benefits from aggressive or moderate treatment and avoid ineffective treatment.

Conventionally, the Cox proportional hazard (CoxPH) model^3^, as represented in Equation (1), evaluates the hazard function of the event occurring at time *t*, *h_i_* (*t*,*z*_i_), for a patient *i* based on the linear combination of the covariates (*Z*), in which *β* is a regression coefficient, *p* is a vector of unknown variables, and *h*_0_(*t*) is an indefinite baseline hazard function.

(1) $$h_{i}\left(t,z_{i}\right)=h_{0}\left(t\right)\prod_{k=1}^{p}e^{\beta_{k}Z_{\mathrm{ik}}}$$

whereas the CoxPH model assumes that each covariate influences patient’s risk factor independent of another covariate, in other words, it presumes a linear combination of covariates, it may be too naive to model the effect of nonlinear risk factors on patient’s survival.

To address these drawbacks, tremendous effort and methods have been employed in survival analysis.

Hitherto, machine learning algorithms such as random forest, artificial neural networks (ANNs), and support vector machines have shown striking results in many applications. Machine learning algorithms also have been effectively adopted, either as competition (e.g., the random survival forest [RSF]^4^) or as a complement (e.g., Cox‐net), with the standard survival analysis model such as CoxPH.^5^

The concept of using an ANN in survival analysis, for learning nonlinear risk functions, was first proposed by the Faraggi‐Simon network.^6^ In this approach, the amount of *βZ_i_* in Equation (1), was replaced with the output of a single‐layer feed‐forward neural network to determine the vector of the unknown parameters *θ*. Though the Faraggi‐Simon model did not significantly outperform the standard CoxPH model, it suggested that a similar extension can be constructed. Since then, many attempts have been performed to acquisition and handle the superb capacity of the neural network in the survival analysis.

Recently, deep learning has attracted remarkable attention for modeling the complex interactions between the covariates in the survival analysis,^7^, ^8^ among them the deep learning‐based survival model (DeepSurv)^9^ has provided striking results. Hitherto, some studies suggested that the DeepSurv models have learned efficiently the complex patient’s risk factor obtained from multiple parameters and outperformed in estimating the failure of treatment for different cancer types such as cervical, oral, and lung cancers.^10^, ^11^ However, to our knowledge, no study has been performed on patients with GBM. Furthermore, the performance of the deep learning‐based survival model is intimately affected by the appropriate configuration of the model hyperparameters. Albeit, it has remained a challenging and time‐consuming task due to computational and process limitations.

On the other hand, given that GBM is a highly heterogeneous tumor at both molecular and histological levels, the combination of both clinical manifestations of the patients and the molecular marker of GBM may give a better survival prediction.^12^ However, rare studies have investigated the combined influence of characteristics such as clinical data, tumor characteristics, treatment options, and molecular markers of GBM in the survival model’s predictive accuracy.^13^ Accurate patients’ survival prediction remains a challenging work.

**FIGURE 1:**
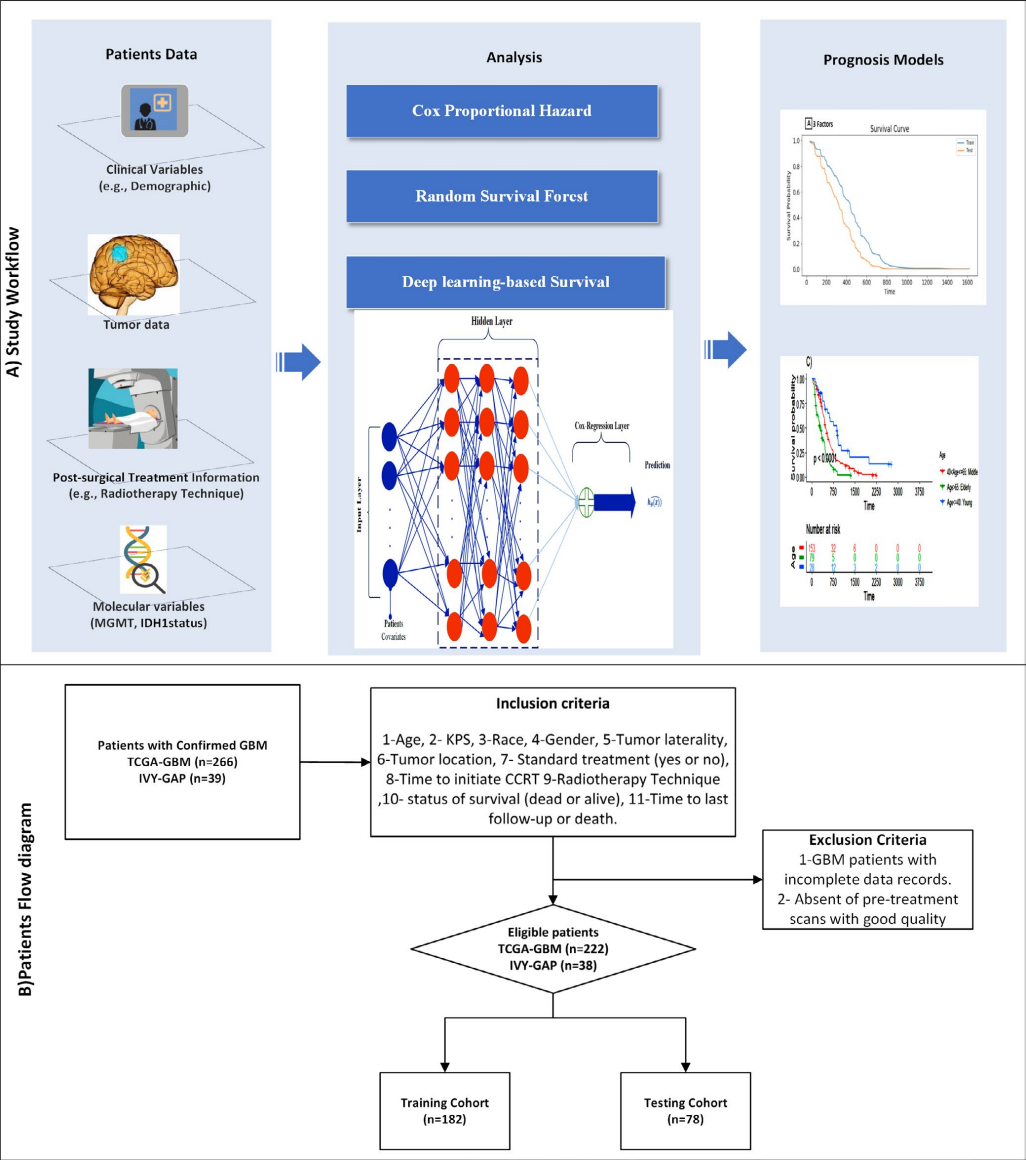
Study workflow and patients flow diagram. Panel (A) workflow of this study. Panel (B) study flow diagram of patients’ recruitment, with the exclusion criteria. CCRT, concurrent chemoradiotherapy

Therefore, this study was designed to investigate three issues: (i) To evaluate the effect of concurrent multivariate risk factors including patient characteristics, tumor site, post‐surgery treatment specification, and molecular marker on patient’s survival; (ii) To validate the deep learning‐based survival model performance in comparison with two other survival reference models (i.e., CoxPH and RSF); and (iii) To assess the impact of hyperparameter optimization methods (random search and Bayesian optimization) on the performance of deep learning‐based survival model. The workflow of our work is summarized in Figure 1A.

The rest of the paper is as follows. In the next section, our dataset, the survival models, particularly the deep learning‐based survival model, are clarified. Furthermore, hyperparameter optimization techniques and statistic metrics used for our assessment are explained in detail. Experimental results are illustrated in Section 3. Finally, a comparison analysis with the results of previous studies and the conclusion of our work is described in Section 4.

## 2 MATERIALS AND METHODS

### 2.1 Data collection

Of the 305 histopathological confirmed GBM patients, 260 cases (163 males and 97 females with a mean age of 59 years) were selected. Patients were eligible who meet our inclusion criteria as follows: (i) demographic characteristics encompass the gender, age at surgery, Karnofsky performance score (KPS), and race; (ii) tumor laterality and location, (iii) detailed of post‐surgical treatment, that is, time to start concurrent chemoradiotherapy (CCRT) after surgery, receiving standard treatment (yes or no); (iv) radiotherapy techniques including three‐dimensional conformal radiotherapy (3D‐CRT), intensity‐modulated radiotherapy (IMRT), other (stereotactic or brachytherapy); and (v) access to last follow‐up time and death status of survival (dead or alive). The flow diagram of patients’ recruitment, with the inclusion criteria in the study, is depicted in Figure 1B.

These data are available from the The Cancer Genome Atlas Glioblastoma Multiforme (TCGA‐GBM) series^14^ and IVY Glioblastoma Atlas Project (Ivy GAP) database^15^ and provides the pathological, genetic, clinical data, and radiological data of patients. Both datasets are without a patient identifier, so the approval of the institutional review board is not required.

The per‐operatives magnetic resonance volumes were employed to determine tumor laterality and tumor location of the selected TCGA‐GBM patients by a specialist. In Table 1, the clinical and tumor information of the eligible cases for each series (TCGA‐GBM and IVY GAP) is summarized.

**TABLE 1:** Demographic information and tumor characteristics of eligible patients (from the TCGA‐GBM and IVY Gap datasets) with glioblastoma

Collection (*n*)	Gender (*n*, %)	Age (*n*, %)	KPS (*n*, %)	Race (*n*, %)	Tumor laterality (*n*, %)	Tumor location (*n*, %)
TCGA‐GBM (221)	Female (80, 36%)	Age < 40 (25, 11%)	KPS ≤ 70 (53, 24%)	White (204, 93%)	Right (103, 47%)	Frontal (64, 29%)
	Male (141, 64%)	40 ≤ Age ˂ 65 (125, 57%)	70 < KPS ≤ 90 (130, 58%)	Black (12, 5%)	Left (118, 53%)	Temporal (63, 28%)
		Age ≥ 65 (71, 32%)	KPS > 90 (38, 18%)	Asian (5, 2%)		Parietal (37, 17%)
						Occipital (19, 9%)
						Other^a^ (38, 17%)
IVY‐GAP (39)	Female (17, 44%)	Age < 40 (3, 7%)	KPS ≤ 70 (7, 18%)	NA	Right (27, 69%)	Frontal (12, 31%)
	Male (22, 54%)	40 ≤ Age < 65 (28,72%)	70 < KPS ≤ 90 (21, 53%)		Left (12, 31%)	Temporal (10, 26%)
		Age ≥ 65 (8, 21%)	KPS > 90 (11, 29%)			Parietal (13, 33%)
						Occipital (3, 8%)
						Other (1, 2%)

Abbreviations: GAP, Glioblastoma Atlas Project; KPS, Karnofsky performance score; NA, not available; TCGA, The Cancer Genome Atlas Glioblastoma Multiforme.

^a^ Other is related to tumors located at other lobes or more than one lobe.

Moreover, isocitrate dehydrogenase 1 (IDH1) and O‐6‐methylguanine‐methyltransferase (MGMT) were integrated into our survival analysis as molecular markers. These markers have frequently been reported as favorable prognostic factors of GBM patients. The molecular characterization of the TCGA‐GBM dataset is available through the Genomic Data Commons Data Portal and was extracted by the Bioconductor (open development and free, open‐source software) packages in R (version 3.6.2) languages. The clinical and genomic data of IVY GAP are accessible via https://glioblastoma.alleninstitute.org. The distribution of molecular markers, that is, IDH1 and MGMT, for all eligible patients is represented in Table S1.

### 2.2 Survival models

Survival analysis (or time‐to‐event analysis) is an actuarial method that has tremendous applications in clinical oncology. One of the main objectives in survival analysis is to designate the probability of occurrence of the event of interest (e.g., death time) beyond any specified time (*t*), that is, survival function *S*(*t*) = Pr(*T* > *t*). Alternatively, the survival function can be accessed by *S*(*t*) = exp(−*H*(*t*)), where *H*(*t*) is the cumulative hazard function (CHF) and is defined as $H\left(t\right)=\int_{0}^{t}h(x)dx$. Heretofore, a wide range of statistical methods has been presented in three main categories (i.e., parametric, non‐parametric, and semi‐parametric) to estimate the survival function and hazard ratio. In this study, the performance of the state‐of‐the‐art survival model, that is, deep learning‐based survival models, is compared alongside the two reference survival models, that is, CoxPH and RSF.

#### 2.2.1 Cox proportional hazard

There is no doubt, that the CoxPH models are the most pervasive survival models in medical analysis, because of their simple execution and informative explanation. As described earlier (Equation 1), the CoxPH model is a linear combination of the covariate (*β*_1_ *Z*_1_+ … +*β*_p_ *Z*_p_). If denote *c_k_* (*c*_1_, … *c_k_*) the possibly censored event time for individual *k*, the corresponding partial likelihood^16^ is defined by Equation (2).

(2) $$L_{\text{Cox}}=\prod_{k=1}^{n}{\left(\frac{e^{\beta_{k}Z_{k}\left(c_{k}\right)}}{\sum_{j\in R_{k}}e^{\beta_{k}Z_{k}\left(c_{k}\right)}}\right)}^{D_{k}}$$

where *R_k_* refers to the set of individuals at risk at event time, and if individual *k* is an observed event time, *D_k_* = 1 otherwise *D_k_* = 0.

#### 2.2.2 Random survival forest

Random survival forest, an extension of Bierman’s random forest method in survival analysis, is a non‐linear and non‐parametric model.^4^ RSF is determined based on an ensemble tree, where a tree is grown by applying B bootstrap samples randomly of each data. Almost 37% of the data are excluded in each bootstrap (in‐bag) sample, which implies out‐of‐bagdata. Subsequently, variables with suitable criteria with maximum log‐rank risk tests are nominated and randomly selected to dichotomize each node of a tree.

This process iteratively is continued until it met the stopping criteria. The ensemble CHF is determined by averaging over the CHF of each tree from nodes with no further split (terminal nodes). Eventually, the prediction error of ensemble CHF is estimated by the concordance index (*c*‐index).

#### 2.2.3 DeepSurv

DeepSurv, a deep feed‐forward neural network, is a non‐linear extension of the CoxPH model.^9^ The risk function (*h_θ_* (*z*)) of DeepSurv is estimated by the network output and is parameterized using the weight of the network (θ). The loss function of the network (Equation 3) is computed by taking a negative log over the partial likelihood of L_cox_ from Equation (2), with extra modification.

(3) $$\text{loss}\left(\theta \right):=‐\frac{1}{n\left(D=1\right)}\sum_{k:D_{k}=1}\log\left(\sum_{j\in R_{k}}\exp\left(\beta_{k}Z_{k}\left(c_{k}\right)\right)‐\beta_{k}Z_{k}\left(c_{k}\right)\right)+\lambda *∥\theta_{2}^{2}∥$$

where *R_k_* refers to the set of individuals at risk at event time, and *D_k_* = 1 if individual *k* is an observed event time, otherwise *D_k_* = 0.

DeepSurv has employed more advanced training methods such as rectified linear unit (ReLU) function, dropout, batch normalization, etc., using TensorFlow (an open‐source Python library) to improve efficiency.

Deep neural networks have demonstrated remarkable performance on many machine learning applications.^17^ However, their performance is highly affected by the appropriate configuration of the model hyperparameters to yield the minimum value of the loss function and the best value for the model parameters. Lately, Bayesian optimization has demonstrated promising results by providing more powerful and intelligent tools for assessing search spaces.^18^ In contrast to random search or grid search, Bayesian optimization is sequential model‐based optimization, in which the mean and variance of the model are sequentially updated to the last observation.^19^

In this work, the CoxPH and the RSF were performed using the R packages, survival, and randomForestSRC, respectively, while DeepSurv by an open‐source Python package. Hyperopt, a TensorFlow Python package,^20^ was employed for Bayesian hyperparameter optimization.

### 2.3 Statistical analysis

Typical metrics such as the root mean squared error are inappropriate for survival model analysis since survival data usually incorporate censoring data. In general, censoring occurs in survival data due to missing patients’ follow‐up or patients alive more than the study time. In this work, variables prognosis was assessed by the univariate and multivariate CoxPH regression model, the log‐rank test, and the hazard ratio. The performance of the survival models was evaluated by the *c*‐index.^21^ Extra information about these metrics is available in Data S1. Survival curves were plotted using the Kaplan–Meier methods. All statistical analysis were performed by the R language (https://www.r‐project.org/, version 3.6.2), using the survival, survminer, and survivalROC packages.

## 3 RESULTS

**FIGURE 2:**
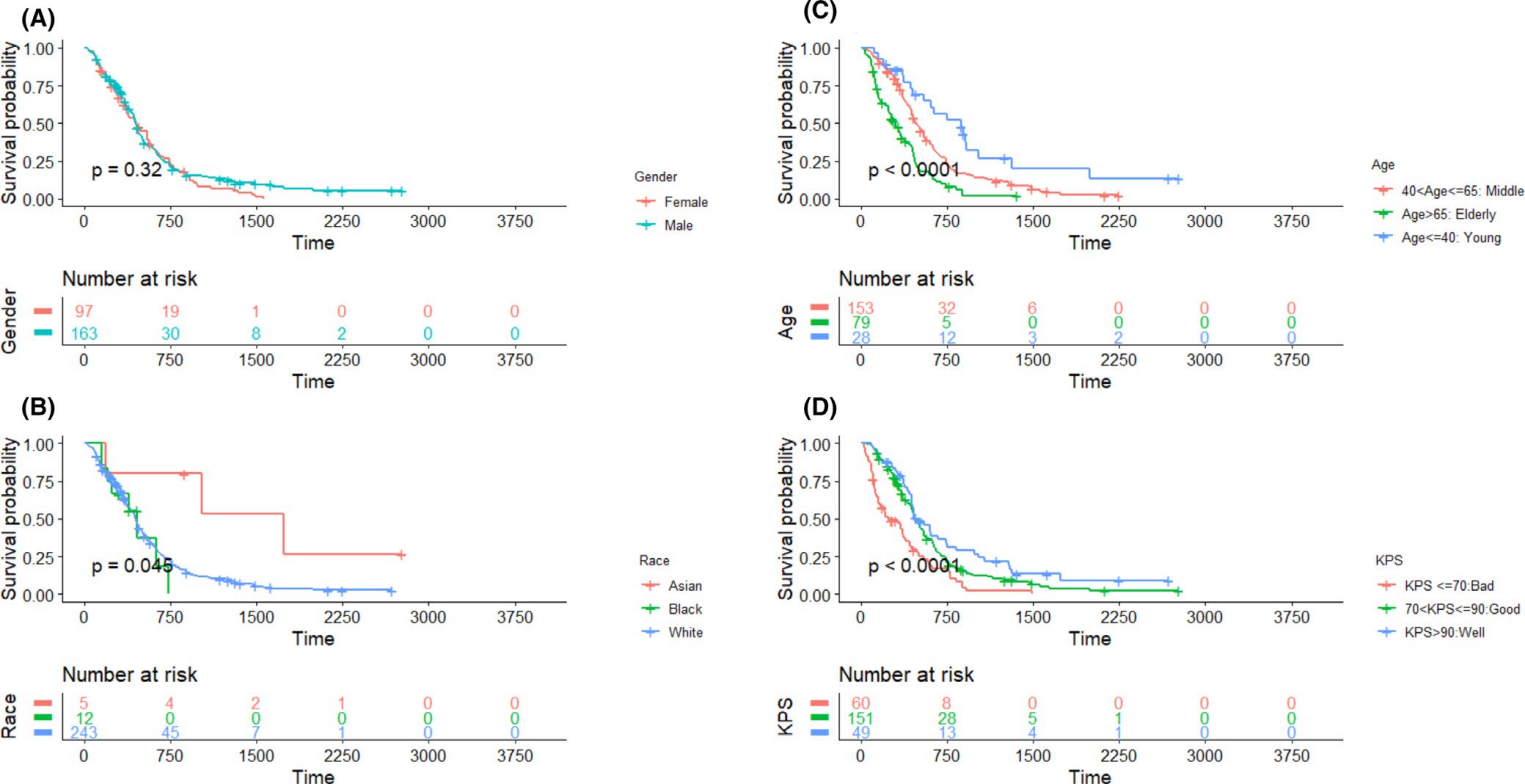
Kaplan–Meier curves on the overall dataset (260 patients) are represented for clinical factors, with pairwise comparisons using the log‐rank test and risk table. Panels (A–C), respectively, confirm that the KPS, age, and race are statistically significant prognoses. While panel (D) shows, gender is not a statistically significant prognostic factor. KPS, Karnofsky performance score

**FIGURE 3:**
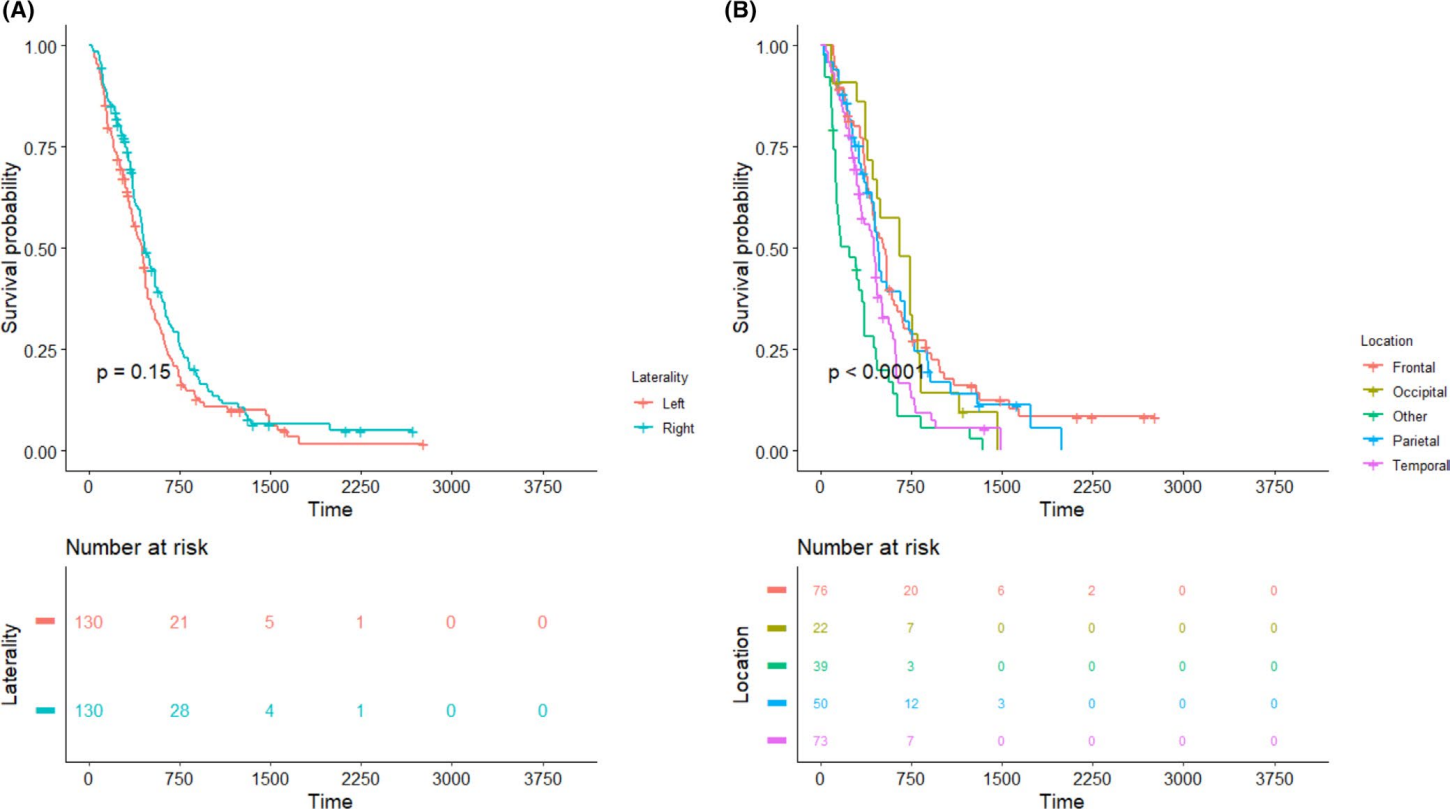
Kaplan–Meier curves on the 260 patients are represented for tumor factors, with pairwise comparisons using the log‐rank test and risk table. (A) Present a statistically significant differences in the log‐rank test for tumor location, but the (B) shows no statistically significant difference in the tumor laterality

**FIGURE 4:**
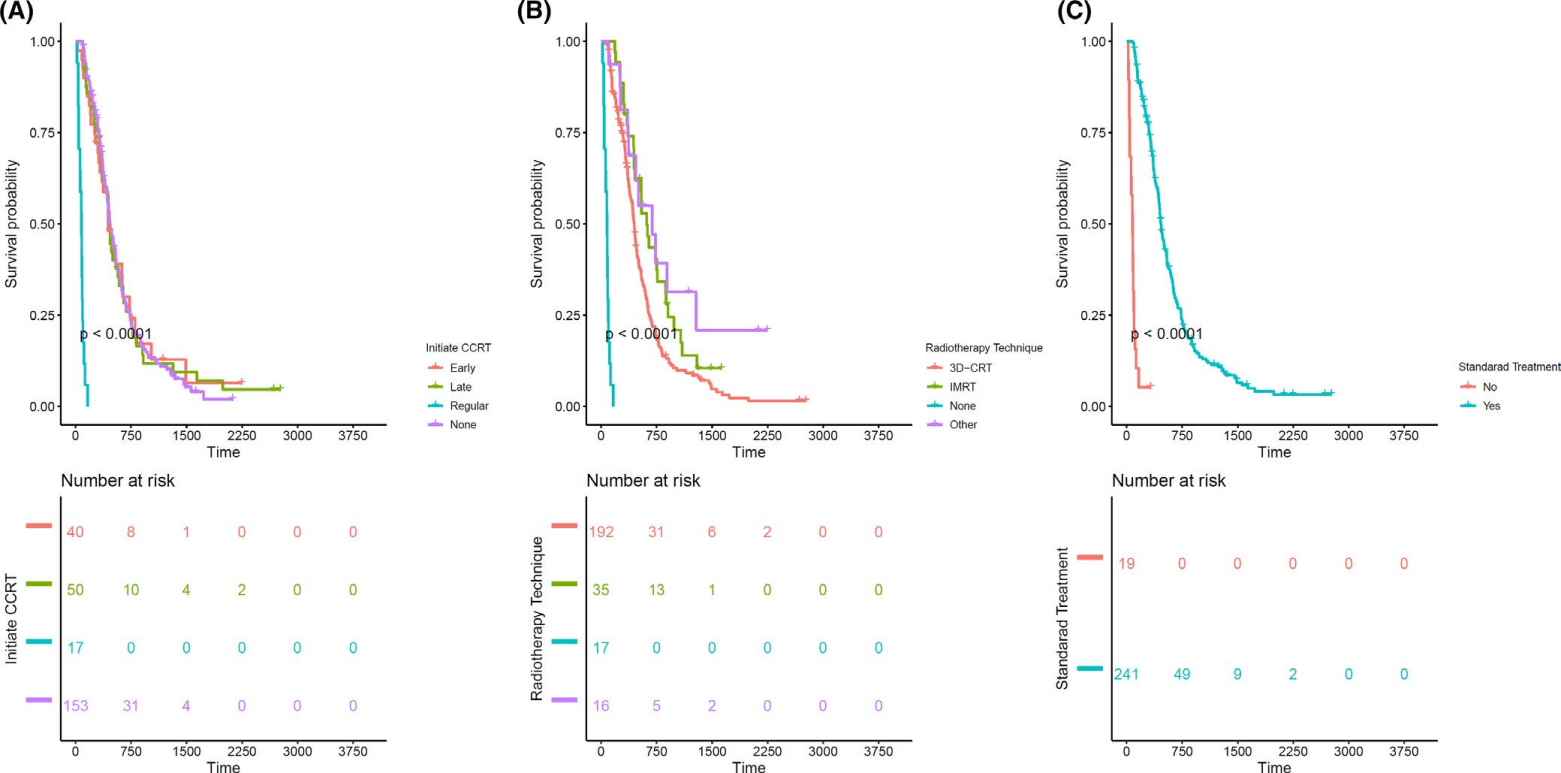
Kaplan–Meier curves on the 260 GBM patients are represented for treatment factors, with pairwise comparisons using the log‐rank test and risk table. (A) Shows a statistically significant difference in the survival of patients who did not start any standard treatment and patients who initiated standard treatment at regular time with a *p* value < 0.0001. (B) Indicate a statistically significant difference in the survival of patients who did not receive any radiotherapy and patients who received radiotherapy with a *p* value < 0.0001. (C) Present a statistically significant difference in the survival of patients receiving standard radiotherapy with concomitant chemotherapy (Commonly temozolomide) compared with those who did not receive such treatment with a *p* value < 0.0001. 3D‐CRT, three‐dimensional conformal radiotherapy; GBM, glioblastoma; I_CCRT, initiate concurrent chemoradiation therapy; IMRT, intensity‐modulated radiation therapy; ST, standard treatment

**FIGURE 5:**
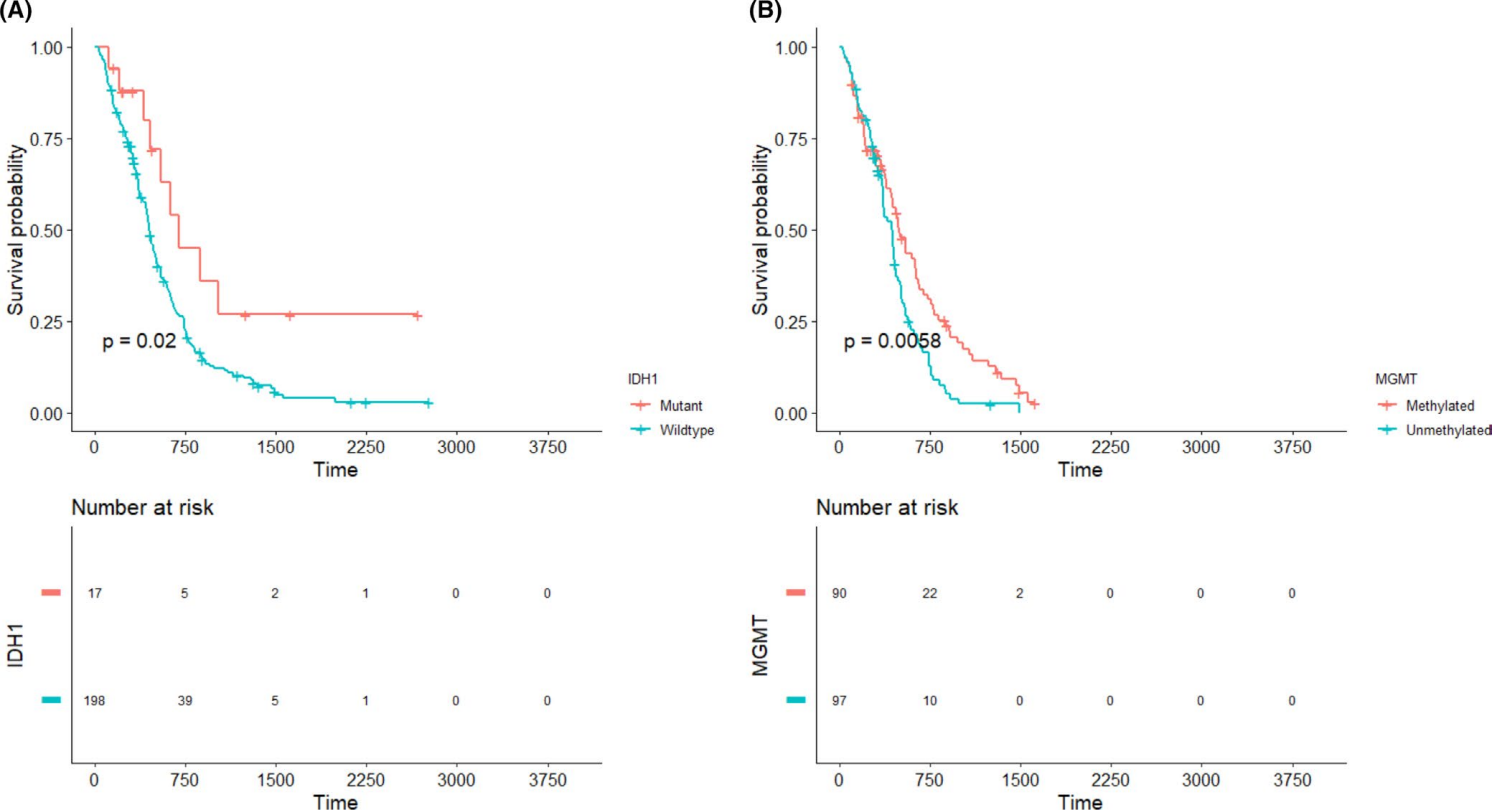
Kaplan–Meier curves on 260 patients are represented for molecular markers (IDH1 and MGMT), using the log‐rank test pairwise comparisons. (A) Indicates that IDH1 (wild‐type) is statistically significantly related to poor prognosis with a *p* value < 0.02. (B) Shows methylated MGMT is related to a better prognosis with a *p* value < 0.00058. IDH1, isocitrate dehydrogenase 1; MGMT, O‐6‐methylguanine methyltransferase

The Kaplan–Meier curves of the demographic information of patients, tumor variables, and post‐surgical treatment characteristics, as well as molecular markers (e.g., IDH1 and MGMT), are presented in Figures 2, 3, 4, 5, respectively, to assess the concurrent prognostic effect of inclusion criteria in this study.

The relevant variables to the prognosis of the GBM patients were rated by univariate CoxPH analysis for both clinical and molecular variables, as are represented in Tables S2 and S3, respectively.

The multivariate analysis was performed using statistically significant variables in univariable analysis (Table 2).

**TABLE 2:** Multivariate analysis over statistically significant univariate variables for overall survival in patients with glioblastoma using Cox proportional hazard regression models with concordance index = 0.69 and log‐rank = 9.412e‐19

Characteristic	Overall survival		
	Factors (*n*)	Hazard ratio (95% CI)	*p* value
Demographic			
Age	40 < Middle ≤ 65 (153)	Reference	
	Elderly > 65 (79)	1.70 (1.229–2.34)	0.018*
	Young ≤ 40 (28)	0.58 (0.343–0.99)	0.034*
KPS	Bad ≤ 70 (60)	Reference	
	70 < Good ≤ 90 (151)	0.96 (0.660–1.39)	0.827
	Well > 90 (49)	0.80 (0.503–1.28)	0.352
Race	Asian (5)	Reference	
	Black (12)	2.93 (0.73–11.74)	0.13
	White (243)	2.92 (0.888–9.57)	0.078
Tumor			
Location	Frontal (76)	Reference	
	Occipital (22)	0.79 (0.471–1.33)	0.381
	Multiple^a^ (39)	1.58 (1.16–2.46)	0.012*
	Parietal (50)	1.06 (0.705–1.60)	0.771
	Temporal (73)	1.25 (0.859–1.81)	0.045
Post‐surgery treatment			
Initiate CCRT	Early (40)	Reference	
	Late (50)	1.03 (0.593–1.60)	0.92
	None (17)	8.84 (0.994–78.68)	0.05
	Regular (153)	0.88 (0.583–1.34)	0.56
Radiotherapy type	3D‐CRT (192)	Reference	
	IMRT (35)	0.68 (0.433–1.035)	0.082
	None (17)	Reference	
	Other^b^ (16)	0.5 (0.266–0.95)	0.035*
Standard treatment	No (19)	Reference	
	Yes (241)	0.35 (0.0469–2.75)	0.32

Abbreviations: 3D‐CRT, three‐dimensional conformal radiation therapy; CCRT, concurrent chemoradiation therapy; CI, confidence interval; IMRT, intensity‐modulated radiation therapy; KPS, Karnofsky performance score.

^a^ Other relates to tumors located at more than one lobe.

^b^ Multiple is related to remaining radiotherapy methods such as stereotactic or brachytherapy.

* Variable with *p* value < 0.05 is considered significant.

Henceforth, the entire eligible dataset was randomly divided into the training set (70%) and into the testing set (30%), which was repeated 10 times to ensure all data were examined.

The survival outcome of the training set (to building the predictive model) and testing set (to assess the prediction model accuracy) was not significantly different. The *c*‐index was computed in each iteration on the train and test datasets. The ultimate *c*‐index for three predictive survival models, that is, CoxPH, RSF, and DeepSurv, was obtained by averaging across indicators.

The optimum value of the hyperparameters was selected using two main strategies which include: (i) random search and (ii) Bayesian optimization. The performance of the hyperparameter tuning was evaluated by *k*‐means cross‐validation (*k* = 5). A configuration with the largest validation *c*‐index was determined to avoid the models’ overfitting. For searching hyperparameters, 100 iterations were performed. The selected hyperparameters used in the configuration of the DeepSurv models are represented in Table S4.

The DeepSurv model was developed with a three‐layer neural network.

**FIGURE 6:**
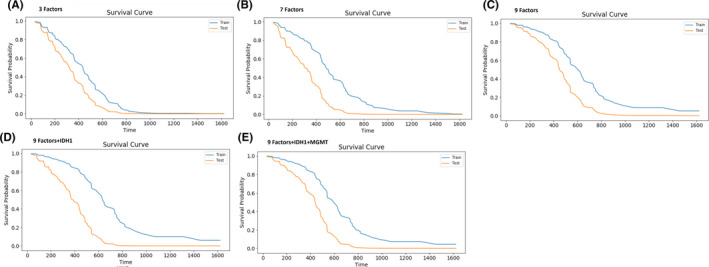
Kaplan–Meier survival curves of the deep learning‐based survival model (DeepSurv, optimized with Bayesian hyperparameter) on the training and testing datasets. Panel (A) shows the survival curve for three statistically significant multivariate factors (age, tumor location, and radiotherapy types), panel (B) shows the survival of seven statistically significant univariate factors (age, tumor location, and radiotherapy types, KPS, race, initiate CCRT, and standard treatment), panel (C) shows the survival curve of all factors (age, tumor location, and radiotherapy types, KPS, race, initiate CCRT, standard treatment, gender, and laterality), panel (D) shows the survival curve of a molecular marker (IDH1) combined with nine factors, and panel (E) shows the survival curve of molecular markers (IDH1 and MGMT) combined with nine factors. CCRT, concurrent chemoradiation therapy; IDH1, isocitrate dehydrogenase 1; KPS, Karnofsky performance score; MGMT

Our analysis was started with three multivariate statistically significant factors (i.e., age, tumor location, and radiotherapy methods). Thereupon, statistically significant variables in univariate analysis, insignificant factors, and molecular markers were integrated into our survival analysis. Kaplan–Meier survival curves of these combinations of risk factors on both training and testing datasets were plotted for the deep learning‐based survival model (DeepSurv) in Figure 6.

A comparison of the performance of survival models, that is, CoxPH, RSF, and DeepSurv, tuning with random search and Bayesian hyperparameter, at each level of increasing the variables is presented in Table 3.

**TABLE 3:** Comparison of the DeepSurv, RSF, and CoxPH survival models by the *c*‐index, with different combinations of variables, on the train and test datasets

Level	Survival model	*c*‐index							
		DeepSurv (Random search)		DeepSurv (Bayesian optimization)		RSF		CoxPH	
	Number of risk factors	Train	Test	Train	Test	Train	Test	Train	Test
1	Three factors (A + Lo + RT)	0.670	0.602	0.672	0.610	0.631	0.597	0.629	0.598
2	Seven factors (A + Lo + RT + KPS + I_CCRT + RT + ST + L)	0.702	0.587	0.72	0.608	0.679	0.592	0.667	0.590
3	Nine factors (A + Lo + I_CCRT + KPS + RT + ST + L + R + G)	0.774	0.631	0.784	0.625	0.698	0.614	0.683	0.612
4	Nine factors + IDH1	0.779	0.638	0.796	0.645	0.701	0.625	0.681	0.610
5	Nine factors + IDH1 + MGMT	0.808	0.693	0.823	0.70	0.728	0.668	0.713	0.674

Abbreviations: CCRT, concurrent chemoradiation therapy; *c*‐index, concordance index; CoxPH, Cox proportional hazard; I_CCRT=initiate CCRT; IDH1, isocitrate dehydrogenase 1; KPS, Karnofsky performance score; MGMT, O‐6‐methylguanine‐methyltransferase; RSF, random survival forest; RT, radiotherapy technique; ST, standard treatment.

A = age, Lo = location, L = laterality, R = race, G = gender.

## 4 DISCUSSION

Though standard treatments of GBM tumors only postpone tumors growth for a while, introducing novel and personalized treatment methods may engender a new door to improve prognosis or even cure this disease. Accurate survival predictions are always desirable for physicians and patients to individualize treatment planning and avoid inessential treatments.

This study was designed to examine the influence of concurrent variables, that is, clinical, tumor, post‐surgery treatment, and molecular factors, and mainly to validate and optimize the performance of deep learning‐based survival models in improving the prediction accuracy of survival models.

Our results in Table S2 indicate that risk factors including race, age, KPS, tumor location, standard treatment, time to initiate CCRT, and type of radiotherapy are univariate significant for overall survival prediction (*p* value ≤ 0.05). However, three of them (i.e., age, tumor location, and type of radiotherapy) were statistically significantly prognosis in multivariate analysis (Table 2). In agreement with previous work,^22^ age and tumor location factors were reported as significant multivariate covariates. Since, study results have demonstrated that univariable relations alone may not be sufficient and informative to determine important significant variables, particularly for complex datasets, using significant univariate statistical variables in multivariate analysis is the most common approach. Multivariate analysis selects the variables that are independently most closely related to prognosis^23^, given that two interrelated variables are unlikely to choose both as significant variables by multivariate analysis.

Our results indicated that younger patients’ survival was better than elderly patients. Though there is no consensus in standardized age cutoff,^24^ we categorized patients into elderly ≥ 65,^25^, ^26^ young < 40,^27^ and middle (40 ≤ age < 65) ages, based on the mentioned references. Age consistently is reported in various literature as efficacious prognostic survival variable,^26^, ^28^, ^32^, ^33^, ^34^.

In consensus with previous works,^29^, ^30^ our findings also showed that tumors located at the temporal lobes or multiple lobes were associated with unfavorable prognostic factors compared with tumors located at frontal lobes. The poor prognosis of tumors located at the temporal lobes can be interpreted by the results of Kocher et al.,^31^ in which tumors located at the temporal lobe and the parietal lobe were determined as the most vulnerable lobes for cognitive function in patients with GBM. Furthermore, patients who received non‐standard treatment of GBM showed the worst prognosis, which is compatible with the benefit of current standard care of GBM patients (i.e., radiotherapy concurrent with chemotherapy, e.g., Temozolomide).

Besides, patients who underwent radiotherapy with stereotactic therapy or brachytherapy were statistically associated with a favorable prognosis. However, only 7% of cases were treated using these methods, and further studies are required to accept this factor as a biomarker. These findings are supported by the fact that ongoing improvements in rmedical imaging and radiation therapy techniques that have facilitated treatment volume delineation and treatment conformality.^32^, ^33^ For example, in contrast to the 3D‐CRT, IMRT feasible further treatment conformality using several modulated beams with various intensities at different angles. Stereotactic radiotherapy allows for an even more accurate representation of treatment volumes while saving surrounding vital structures, using many beam sources

To build survival models, we started with three significant variables in our multivariate analysis. At this step, the DeepSurv (optimized with random search), CoxPH, and RSF models achieved a *C*‐index of 0.67, 0.629, and 0.631, respectively. Subsequently, all variables were added in five levels to evaluate the effect of different variables combinations on survival model accuracy. After involving all variables in the analysis (fifth level), the DeepSurv models configured with random search (*c*‐index = 0.808) outperformed the CoxPH (*c*‐index = 0.713) and RSF (*c*‐index = 0.728) survival models. Furthermore, at this level, the DeepSurv model, configured with Bayesian hyperparameter optimization, strikingly performed best among the three survival models and achieved the highest *c*‐index = 0.823.

In conclusion, from our promising findings, four analytical issues were deduced. First, age, tumor location, and methods of radiotherapy were independently significant prognosis variables. Second, even insignificant prognosis variables played roles in improving the predictive accuracy of survival models.

Third, the deep learning‐based survival model outperformed the Cox proportional hazard regression and random survival forest models’ ability for accurate GBM survival prediction. Furthermore, the optimum hyperparameter tuning may remarkably improve the deep learning‐based survival models performance. As a result, the remarkable performance of DeepSurv indicates the ability of the deep learning model in learning complex association of risk factors. The deep learning‐based survival model may have a great potential to be incorporated into the treatment planning of patients with GBM in a routine oncology workflow by improving the prediction of mortality risk of GBM patients. In the future, this work will be extended to investigate the adding value of the reproducible radiomics features to the deep learning‐based survival model.^34^

## CONFLICT OF INTEREST

There is no conflict of interest declared in this article.

## Supporting information

Table S1

Table S2

Table S3

Table S4

Supplementary Material

## Data Availability

The data might be made available upon request, and some restrictions will apply.
