# Supplementary material for: The role of deep learning‐based survival model in improving survival prediction of patients with glioblastoma
Source: Cancer Med. 2021 Aug 28;10(20):7048–59. doi: 10.1002/cam4.4230 (PMC8525162; doi:10.1002/cam4.4230)
Supplement: Supplementary file 1 — Table S1 [file CAM4-10-7048-s004.docx]

Table S1. Distribution of the molecular marker (IDH1 and MGMT) of all eligible patients in TCGA-GBM and IVY GAP datasets.

| Collection | IDH1 (n, %) |  | MGMT (n, %) |
| --- | --- | --- | --- |
| **TCGA-GBM (221)** | Wild-type (164, 74%) |  | Methylated (75, 34%) |
|  | Mutant (13, 6%) |  | Unmethylated (74, 33%) |
|  | Unknown (44, 20%) |  | Unknown (111, 50%) |
| **IVY GAP (39)** | Wild-type (34, 87%) |  | Methylated (15, 38%) |
|  | Mutant (4, 10%) |  | Unmethylated (23, 59%) |
|  | Unknown (1, 3%) |  | Unknown (1, 3%) |

Abbreviation: IDH1=Isocitrate dehydrogenase 1, MGMT=O^6^-methylguanine-methyltransferase.
