# Supplementary material for: The role of deep learning‐based survival model in improving survival prediction of patients with glioblastoma
Source: Cancer Med. 2021 Aug 28;10(20):7048–59. doi: 10.1002/cam4.4230 (PMC8525162; doi:10.1002/cam4.4230)
Supplement: Supplementary file 2 — Table S2 [file CAM4-10-7048-s002.docx]

Table S2. Univariate analysis of the clinical covariates including demographic, tumor, and post-surgery treatment on the overall survival of all patients (260) with glioblastoma by Cox proportional hazard regression models.

|  | Characteristics | Factors (n) | Hazard Ratio (95% CI) | P-value |
| --- | --- | --- | --- | --- |
| **Demographic** | **Age** | 40<Middle ≤65 (153) | Reference |  |
|  |  | Elderly>65 (79) | 2.03 (1.51-2.72) | <0.001^*^ |
|  |  | Young ≤40 (28) | 0.53 (0.33-0.86) | 0.012* |
|  | **KPS** | Bad≤70 (60) | Reference |  |
|  |  | 70<Good≤90 (151) | 0.53(0.39-0.73) | 0.001^*^ |
|  |  | Well>90 (49) | 0.4 (0.26-0.60) | 0.001^*^ |
|  | **Gender** | Female (97) | Reference |  |
|  |  | Male (163) | 0.87(0.66-1.1) | 0.319 |
|  | **Race** | Asian (5) | Reference |  |
|  |  | Black (12) | 4.5 (1.2-17) | 0.028^*^ |
|  |  | White (243) | 3.8(1.2-12) | 0.023^*^ |
| Tumor | **Laterality** | Left (130) | Reference |  |
|  |  | Right (130) | 0.82 (0.63-1.1) | 0.147 |
|  | **Location** | Frontal (76) | Reference |  |
|  |  | Occipital (22) | 1 (0.62-1.7) | 0.897 |
|  |  | Parietal (50) | 1.1(0.74-1.6) | 0.662 |
|  |  | Temporal (73) | 1.6 (1.12-2.3) | 0.01^*^ |
|  |  | ^§^Multiple (39) | 2.6 (1.73-3.9) | <0.001^*^ |
| **Post-surgery Treatment** | **Initiate CCRT** | Early (40) | Reference |  |
|  |  | Late (50) | 0.99 (0.63-1.6) | 0.971 |
|  |  | None (17) | 41.17(19.98-87) | <0.001^*^ |
|  |  | Regular (153) | 1.02 (0.69-1.5) | 0.935 |
|  | **Radiotherapy Type** | EBRT (192) | Reference |  |
|  |  | IMRT (35) | 0.64 (0.43-0.96) | 0.029^*^ |
|  |  | None (17) | 37.22 (19.40-73.35) | <0.001^*^ |
|  |  | ^ǂ^ Other (16) | 0.49 (0.26-0.9) | 0.021 |
|  | **Standard Treatment** | No (19) | Reference |  |
|  |  | Yes (241) | 0.046 (0.026-0.081) | <0.001^*^ |
