# Supplementary material for: The role of deep learning‐based survival model in improving survival prediction of patients with glioblastoma
Source: Cancer Med. 2021 Aug 28;10(20):7048–59. doi: 10.1002/cam4.4230 (PMC8525162; doi:10.1002/cam4.4230)
Supplement: Supplementary file 3 — Table S3 [file CAM4-10-7048-s005.docx]

Table S3. Univariate analysis of molecular Characteristics on the overall survival of all patients (260) with glioblastoma by Cox proportional hazard regression models.

| Molecular Characteristics | Factors (n) |  | Hazard Ratio (95% CI) | P-value |
| --- | --- | --- | --- | --- |
| **IDH1** | Mutant (17) |  | Reference |  |
|  | Wild-Type (198) |  | 2.2(1.1-4.3) | 0.023^*^ |
| **MGMT** | Methylate (90) |  | Reference |  |
|  | Unmethylated (97) |  | 1.6(1.1-2.2) | 0.006^*^ |

*Variable with P-value<0.05 considers significant. CI= Confident interval, c-index= Concordance-index, IDH1=Isocitrate dehydrogenase 1, MGMT=O^6^-methylguanine-methyltransferase.
