# Supplementary material for: The role of deep learning‐based survival model in improving survival prediction of patients with glioblastoma
Source: Cancer Med. 2021 Aug 28;10(20):7048–59. doi: 10.1002/cam4.4230 (PMC8525162; doi:10.1002/cam4.4230)
Supplement: Supplementary file 4 — Table S4 [file CAM4-10-7048-s001.docx]

Table S4. Hyperparameters used for the DeepSurv model, optimized with random search and Bayesian hyperparameter optimization.

| Hyperparameter Optimizer | LR | LR-decay |  | Activation | L1-reg | L2-reg | dropout |
| --- | --- | --- | --- | --- | --- | --- | --- |
| **Random search** | 0.7 | 0.89 |  | Tanh | 9.4e-3 | 6.8e-4 | 1 |
| **Bayesian Optimization** | 0.9 | 1 |  | Tanh | 6.98e-5 | 7.7e-5 | 0.9 |

Abbreviation: LR= Learning rate, LR-decay= Learning rate-decay.
