# Supplementary material for: The role of deep learning‐based survival model in improving survival prediction of patients with glioblastoma
Source: Cancer Med. 2021 Aug 28;10(20):7048–59. doi: 10.1002/cam4.4230 (PMC8525162; doi:10.1002/cam4.4230)
Supplement: Supplementary file 5 — Supplementary Material [file CAM4-10-7048-s003.docx]

**The Role of Deep Learning-based Survival Model in Improving Survival Prediction of Patients with Glioblastoma (GBM)**

Hajar Moradmand^1^, Seyed Mahmoud Reza Aghamiri^1^, Reza Ghaderi^2^, Hamid Emami^3^

^1^ Shahid Beheshti University, Medical radiation Engineering, Tehran, Iran

^2^ Shahid Beheshti University, Electrical Engineering, Tehran, Iran

^3^Isfahan University of Medical Sciences, Department of Radiation Oncology

Seyed Al-Shohada Charity Hospital, Isfahan, Iran

Correspondence

Hajar Moradmand

Shahid Shahriari Square, Daneshjou Boulevard, Shahid Chamran Highway, Tehran, 1983969411, Iran

Phone/Fax: (+98)29904125

Email: h_moradmand@sbu.ac.ir

This supporting information has been provided by to give readers additional information.

Supporting Information

## Statistical analysis

The Concordance-index (c-index)^1^ is the generalized measurement of the area under the ROC curve in survival analysis for quantifying the predictive accuracy of the survival model in the censored data. The survival model’s prediction accuracy implies two aspects, the reliability, and the discriminating. The discrimination ability is the key component in the assessment of risk prediction score, and it reflects the capacity of a survival model to provide a reliable ranking between the samples (i.e., log hazard ratio) with different event times. The c-index represent the relative risk scores of every independent pair of patients rather than times-to-event as succinctly is expressed in Equation 4, where $p_{i}$ and $p_{j}$ are referred to the i^th^ and j^th^ patient, respectively, $\zeta$is denoted to the risk score for the ith patient, T is the time to event, and $\gamma$ is the auxiliary variable to identify if the data is censored ($\gamma_{i}=0$) or not ($\gamma_{i}=1$).

|  | $C_{I}=\frac{\sum_{p_{i}\neq p_{j}} I\left\{ \zeta_{p_{i}}<\zeta_{p_{j}} \right\}I\left\{ T_{p_{i}}>T_{p_{j}} \right\}\gamma_{p_{j}}}{\sum_{p_{i}\neq p_{j}} I\left\{ T_{p_{i}}>T_{p_{j}} \right\}\gamma_{p_{j}}}$ | (4) |
| --- | --- | --- |

The range of c-index is from 0 to 1, the c-index closer to 1 indicates the better power of the predictive model.

To compare the survival curves of two or more groups of patients (e.g., high risk and low-risk groups) the log-rank test is used, that assess if the differentiate between two survival function are significantly enough to reject the null hypothesis ($H_{0}\left( t \right): S_{1}\left( t \right)=S_{2}\left( t \right); t=\left\{ t_{1}, t_{2},t_{3},\cdots,t_{k} \right\}$). The log-rank test is distributed and calculated like a chi-square $\left( \chi^{2} \right)$ statistic as is described by a simple formulate in Equation 5, where O and E, respectively, are referred to the total number of observed event and expected to fail in group k, and v is the variance.

|  | $\chi_{( log-rank)}^{2}=\frac{\left( \sum_{k} \left( O_{k}\left( t_{k} \right)-E_{k}\left( t_{k} \right) \right) \right)^{2}}{\sum_{k} v_{k}}$ | (5) |
| --- | --- | --- |

With regard to censored data and null hypothesis in calculating the log-rank test, the expected and variance number of the observed event of group 1 can be defined as $E_{k}=R_{1k}\cdot\frac{d_{k}}{R_{k}}$ , and $v_{k}=\frac{R_{1k}R_{2k}\cdot d_{k}\left( R_{k}-d_{k} \right)}{\left[ {R_{k}}^{2}\left( R_{k}-1 \right) \right]}$, respectively, thereupon the Equation 5 can be rewriting as is represented in Equation 6. Where $d_{1k}$,$d_{2k}$ and $R_{1k}$,$R_{2k}$ are respectively referred to the number of observation failure event and the number of sample at risk in group 1 and group 2 at each time interval ($t=\left\{ t_{1}, t_{2},t_{3},\cdots,t_{k} \right\}$)).

|  | $\chi_{( log-rank)}^{2}=\frac{\left( \sum_{k} \left( d_{1k}-R_{1k}\cdot\frac{d_{k}}{R_{k}} \right) \right)^{2}}{\sum_{k} {R_{1k}R_{2k}\cdot d_{k}\left( R_{k}-d_{k} \right)}/\left[ {R_{k}}^{2}\left( R_{k}-1 \right) \right]}$ | (6) |
| --- | --- | --- |

Hazard ratio (HR) is frequently used to estimate the relative risk of the effectiveness of risk factors on the time-to-event, is calculated based on the CoxPH model, and selected based on significant P-value. HR can be formulated as represented in Equation 7, an increase in HR value (in other words, HR>1) relative to the referenced group expresses a worse prognosis, whereas the HR<1 shows a better prognosis in contrast to the referenced group.

|  | $HR=\frac{\sum_{k} {O_{k}}/{E_{k}}}{\sum_{j} {O_{j}}/{E_{j}}}$ | (7) |
| --- | --- | --- |

Reference

**1.** Harrell FE, Jr., Lee KL, Califf RM, Pryor DB, Rosati RA. Regression modelling strategies for improved prognostic prediction. *Stat Med.* Apr-Jun 1984;3(2):143-152.
